# Editorial: Advances and Trends in Development of Plant Factories

**DOI:** 10.3389/fpls.2016.01848

**Published:** 2016-12-09

**Authors:** Alejandro I. Luna-Maldonado, Juan A. Vidales-Contreras, Humberto Rodríguez-Fuentes

**Affiliations:** Department of Agricultural and Food Engineering, Faculty of Agriculture, Autonomous University of Nuevo LeonNuevo Leon, Mexico

**Keywords:** plant factories, intelligent systems, environment controlled and optimization, pharmaceuticals, genetic engineering, biofertilizers

The plant factory is a facility that aids the steady production of high-quality vegetables all year round by artificially controlling the cultivation environment (e.g., light, temperature, humidity, carbon dioxide concentration, and culture solution), allowing growers to plan production. By controlling the internal environment, plant factories can produce vegetables about two to four times faster than by typical outdoor cultivation. In addition, as multiple cultivation shelves (a multi-shelf system) are used, the mass production of vegetables in a small space is facilitated.

This research topic presents some new trends on intelligent measuring systems; environment controlled and optimization; flavonoids; phenylpropanoids, transcriptomes, and bacteria.

Among some of the new findings on intelligent measuring systems, Chen et al. developed an automated measurement system to measure and record the plant weight during plant growth in plant factory. They found that plant weights measured by the weight measurement device are highly correlated with the weights estimated by the stereo-vision imaging system. Moriyuki and Fukuda devised a novel high-throughput diagnosis system using the measurement of chlorophyll fluorescence forming an image of 7200 seedlings acquired by a CCD camera and an automatic transferring machine. They used machine learning in order to extract biological indices and predict plant growth. Previously, Hashimoto et al. (2001) applied an intelligent control system consisting of a decision system based on neuronal networks and genetic algorithms to optimize the growth of hydroponic tomato plants during the seedling stage. Hwang et al. (2014) also proposed a plant factory automatic control system to collect crop image information (illuminance, temperature, humidity, EC, pH, and CO2) and provided crop environment control and service suitable for crop growth step (crop shape, size, color, and length).

Related to environment controlled and optimization, Zheng et al. explored the effects of different density treatments on potato spatial distribution and yield in spring and fall. They concluded that increased density significantly increased potato yield, but the degree of influence associated with different growing seasons differed slightly. In Japan, there were 165 plant factories with artificial lighting (Kozai et al., 2015). Wang et al. added an air exchanger for cooling in plant production systems with artificial light (PPAL). They found that using air exchanger to introduce outdoor cold air is as an effective way to reduce electric-energy consumption with little effects on plant growth in a PPAL. Besides, the cultivation methods in a plant factory with artificial lighting (PFLA) were studied by Zhang et al., retarded senescence of outer leaves of lettuce and found white LEDs are more appropriate for lettuce growth than red or blue LEDs. In another research, Li et al. applied LED lighting [LED with zoom lenses (Z-LED) and conventional non-lenses LED (C-LED)]. The improvement saved over half of the light source electricity, while the temperature and rate of photosynthesis abruptly decreased, causing reductions in plant yield and nitrate content, while having no negative effects on morphological parameters and photosynthetic pigment contents. About nighttime supplemental LED inter-lighting, Tewolde et al. found nighttime LED inter-lighting can effectively improve tomato plant growth and yield with lower energy cost compared with daytime both in summer and winter. Moreover, Cao et al. achieved that tomato inside of a solar greenhouse increased their fresh weight with the increase of RL NB frequencies. On the other hand, Wang et al. found that leaf photosynthetic capacity and photosynthetic rate increased with decreasing Red/Blue lights ratio until 1. However, shoot dry weight increased with increasing Red/Blue lights ratio with the greatest value under Red/Blue = 12 treatment. They concluded that quantitative Blue light could promote photosynthetic performance or growth by stimulating morphological and physiological responses. Miyagi et al. (2017) found that synergistic effects of monochromic LED combined with high CO_2_ and nutrients would be beneficial alternatives for cultivation of lettuce in the plant factory. Shimokawa et al. (2014) concluded that simultaneous red and blue irradiation promote plant growth more effectively than monochromatic and fluorescent light irradiation. They also found that alternating red and blue light accelerated plant growth significantly even when the total light intensity per day was the same as with simultaneous irradiation. The fresh weight of the plant doubles compared to normal LED cultivation methods. The plant factory can increase harvests and sales using this this method (Showa, 2016).

The production of pharmaceuticals in a plant factory represents an opportunity for health benefits from plants. Flavonoids are a group of plant metabolites thought to provide health benefits through cell signaling pathways and antioxidant effects (Spencer, 2016). Dong et al. studied soluble flavonoids and found that “Xiangfen 1” banana can be a rich source of natural antioxidants in human diets. Jeong et al. (2015) characterized the polyphenolic contents of lettuce leaves grown under different night-time temperatures and cultivation. Plant-derived phenylpropanoids (PPPs) compose the largest group of secondary metabolites produced by higher plants (Korkina et al., 2011). Dwivedi et al. reviewed the progress for accessing variation in PPPs in germplasm collections. PPPs are a diverse chemical class with immense health benefits that are biosynthesized from the aromatic amino acid L-phenylalanine.

Genetic engineering of plants deals on the creation of plants to resist herbicides and pests, but also to improve the quality of the crops in terms of consumers (Sévenier et al., 2002). Xing et al. found complex regulatory mechanisms involved in floral induction, flower bud formation, and flowering characteristics, which might reflect the genetic variation of the flowering gene and their data provided a foundation for the further exploration of apple diversity and gene–phenotype relationships, and for future research on molecular breeding to improve apple and related species. Higashi et al. studied circadian oscillation of the lettuce transcriptome under constant light and light–dark conditions and found gene expression pattern is related to photosynthesis and optical response performs normally in lettuce. Higashi et al. detected diurnal variation of tomato transcriptome through the molecular timetable method in a sunlight-type plant factory. They found circadian clock mediate the optimization for fluctuating environments in the field and it has possibilities to enhance resistibility to stress and floral induction by controlling circadian clock through light supplement and temperature control. Tanigaki et al. (2016) in their research suggested that the regulation of gating stomata does not depend predominantly on TOC1 and significantly reflects the extracellular environment. Besides, Zhang et al. studied transcriptome analysis of *Dendrobium officinale* and its application to the identification of genes associated with polysaccharide synthesis valuable clues for identifying candidate genes involved in polysaccharide biosynthesis and elucidating the mechanism of polysaccharide biosynthesis. On the other hand, Hiwasa-Tanase and Ezura reviewed on molecular breeding to create optimized crops: From genetic manipulation to potential applications in plant factories and found that Cost-effectiveness is improved from the use of cultivars that are specifically optimized for closed system cultivation. Lai et al. studied two LcbHLH transcription factors interacting with LcMYB1 in regulating late structural genes of anthocyanin biosynthesis in *Nicotiana and Litchi chinensis* during anthocyanin accumulation and found LcbHLH1 and LcbHLH3 are essential partner of LcMYB1 in regulating the anthocyanin production in tobacco and probably also in litchi. The LcMYB1-LcbHLH complex enhanced anthocyanin accumulation may associate with activating the transcription of DFR and ANS.

Bacterial biofertilizers can improve plant growth through several different mechanisms: (i) the synthesis of plant nutrients or phytohormones, which can be absorbed by plants, (ii) the mobilization of soil compounds, making them available for the plant to be used as nutrients, (iii) the protection of plants under stressful conditions, thereby counteracting the negative impacts of stress, or (iv) defense against plant pathogens, reducing plant diseases or death (García-Fraile et al., 2015). Kifle and Laing isolated and screened bacteria for their diazotrophic potential and their influence on growth promotion of maize seedlings in greenhouses. They identified that isolates showed significant effect on at least two growth parameters were at species or genera level.

## Author contributions

All authors listed have made substantial, direct, and intellectual contribution to the work, and approved it for publication.

## Funding

AL acknowledges support from PAYCIT UANL 2015. JV acknowledges support from CONACYT. HF acknowledges support from Administration of Faculty of Agriculture, Autonomous University of Nuevo Leon.

### Conflict of interest statement

The authors declare that the research was conducted in the absence of any commercial or financial relationships that could be construed as a potential conflict of interest.
